# Development of Three-Dimensional Dental Scanning Apparatus Using Structured Illumination

**DOI:** 10.3390/s17071634

**Published:** 2017-07-15

**Authors:** Jae Sung Ahn, Anjin Park, Ju Wan Kim, Byeong Ha Lee, Joo Beom Eom

**Affiliations:** 1 Medical Photonics Research Center, Korea Photonics Technology Institute (KOPTI), Gwangju 61007, Korea; jaesung.ahn@kopti.re.kr (J.S.A.); anjin.park@kopti.re.kr (A.P.); 2 Department of Biomedical Science and Engineering, Gwangju Institute of Science and Technology (GIST), Gwangju 61005, Korea; scienc2@gist.ac.kr; 3 School of Information and Communications, Gwangju Institute of Science and Technology (GIST), Gwangju 61005, Korea; leebh@gist.ac.kr

**Keywords:** 3D intraoral scanner, structured illumination, liquid lens, 3D point cloud

## Abstract

We demonstrated a three-dimensional (3D) dental scanning apparatus based on structured illumination. A liquid lens was used for tuning focus and a piezomotor stage was used for the shift of structured light. A simple algorithm, which detects intensity modulation, was used to perform optical sectioning with structured illumination. We reconstructed a 3D point cloud, which represents the 3D coordinates of the digitized surface of a dental gypsum cast by piling up sectioned images. We performed 3D registration of an individual 3D point cloud, which includes alignment and merging the 3D point clouds to exhibit a 3D model of the dental cast.

## 1. Introduction

Due to its non-destructiveness and speed, non-contact optical three-dimensional (3D) shape measurement has proven to be useful for a myriad of applications, such as obstacle detection for vehicle guidance, dimensional measurement for quality inspection in automated manufacturing systems, and human body scanning [1,2,3,4,5,6,7,8,9,10,11,12,13,14]. Various optical techniques, including the time-of-flight method, triangulation, stereography, confocal microscopy, interferometry, and fringe projection have been developed for measuring 3D shapes [15,16,17,18,19,20].

Among the various applications, the medical applications of non-contact optical 3D shape measurement [8,9,10,11,12,13] have attracted much attention due to its advantages over conventional shape measurement methods using custom moulds [21,22,23]. In particular, there has been enormous interest in the development of a 3D shape measurement apparatus for dental applications. The development of a 3D intraoral scanning apparatus, which takes a digital impression of the teeth rather than a conventional impression, was one of the central parts of research aimed at dental applications [24]. Since the introduction of digital impressions in the late 1980s, there were various commercial attempts to use an intraoral scanning apparatus for obtaining the digital impressions of the teeth [25,26,27,28]. However, there are still technical problems regarding the accuracy of the digital impressions and the scanning speed of the scanning apparatus. Recently, it was reported that a 3D intraoral scanning apparatus using optical sectioning with structured illumination provides wide-field, high-resolution images compared to other 3D intraoral scanning apparatuses [29,30]. Due to the advantages of structured illumination, a 3D intraoral scanning apparatus using optical sectioning with structured illumination seems to facilitate the faster and more accurate acquisition of the digital impressions. For depth scanning, however, 3D intraoral scanning apparatuses based on structured illumination experience the mechanical movement of a focusing lens that needs additional motor-driven actuators [31]. Recently, an electrically tunable liquid lens was used for 3D imaging, 3D microscopy, and 3D orbital tracking because of its fast response and compactness [32,33,34].

In this paper, we demonstrate a 3D intraoral scanning apparatus based on structured illumination. We reduced the mechanical movements of optics inside the 3D dental scanning apparatus by using a piezo stage and a liquid lens, which replaced the motor-driven actuators of conventional scanning apparatuses. Moreover, we made the scanning apparatus more compact by replacing the motor-driven actuators. We recorded 2D images of the dental cast (gypsum teeth model) with varying the focus along the focal axis. For each focus, three consecutive images were captured with laterally shifting the structured illumination. We performed optical sectioning with structured illumination and reconstructed a 3D point cloud, which represents the 3D coordinates of the digitized surface, of each tooth by stacking up the sectioned images along the focal axis. In addition, we performed 3D registration (3D model aligning and stitching) of the 3D point clouds of each tooth to build a 3D model of the dental cast.

## 2. Methods

**Figure 1:**
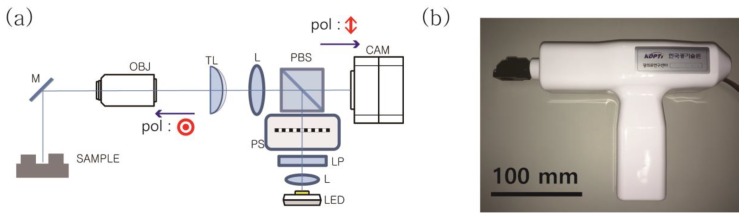
(**a**) Schematic of the experimental setup. The structured light pattern, which was generated by a Ronchi ruling, focused on the sample. The scattered light from the sample was collected by an objective lens and recorded by a camera. The ⊙ and arrow signs (red) represent the polarization of illuminated and scattered light, respectively. (M: mirror, OBJ: objective lens, TL: tunable lens, L: lens, PBS: polarizing beam splitter, CAM: camera, PS: piezo stage, LP: linear polarizer). (**b**) Picture of the three-dimensional scanning apparatus. The dimensions of the apparatus are 275 mm × 176 mm × 72 mm. Scale bar: 100 mm.

Figure 1a shows a schematic of the 3D scanning apparatus. With the exception of the mirror tip (M), the experimental setup was sealed with a housing to block external noise (Figure 1b). The dimensions of the 3D scanning apparatus were 275 mm × 176 mm × 72 mm. We used collimated white light from a light emitting diode (LED) (MCEP-CW8-070-3, Moritex, Saitama, Japan) as an illumination source. A linear polarizer was used to select the vertically polarized component of the illumination source. The collimated beam was illuminated to a Ronchi ruling (1" × 1" (20 lp/mm), Ronchi Ruling #58-777, Edmund Optics, Barrington, NJ, USA) of 50 μm periodicity. The distance between the imaging lens and the Ronchi ruling was the same as the distance between the imaging lens and the camera. The structured light was reflected off a polarization beam splitter (PBS) and traversed through an imaging lens (L) (f = 50 mm), a tunable lens (TL) (EL-10-30-C, Optotune AG), and a long working distance objective lens (OBJ, working distance: 55 mm). The structured light was projected onto the sample surface and the overlapped images of the structured light and the sample were recorded by a CMOS (Complementary metal–oxide–semiconductor) camera (frame rate: 170 fps, MQ022MG-CM, Ximea, Münster, Germany). It is notable that the cross polarization detection technique was used to remove internal reflection from the beam splitter and to enhance the contrast ratio of the recorded images. We used a focus tunable lens, instead of a combination of a solid lens and a mechanical actuator, to change the focus of the objective lens along the direction parallel to the optical axis. The tunable lens was capable of tuning the working distance of the objective lens from 30 to 130 mm by applying a current of 0 to 250 mA in increments of 2.5 mA. We verified the linearity and the axial step of tuning of the working distance by imaging a depth-of-field target (Depth of Field Target 5-15 #54-440, Edmund Optics). The axial step of the tuning of the working distance was 100 μm. It was advantageous that the focus of the tunable lens could be quickly tuned, within a few milliseconds. For each focal length, we took three consecutive images of the sample by translating the Ronchi ruling along the direction perpendicular to the optical axis. The Ronchi ruling was translated by the piezo stage (Q-522, Physik Instrumente, Karlsruhe, Germany) in steps of (50/3) μm across (100/3) μm. We recorded 300 images for the reconstruction of the 3D models at a single position. We used a gypsum dental cast sample (gypsum teeth model) for the reconstruction of the 3D point cloud model and 3D registration of the individual point cloud models. For better quantification of the overlapped region of the adjacent scanning area, we realigned the gypsum dental cast along a straight line. At present, the scanning speed (~1.9 s) for our 3D scanning apparatus is insufficient for hand-held operation, because it is restricted by the sensitivity and speed of the CMOS camera. The sample was mounted on a linear motorized translation stage (M-ILS200CC, Newport, Irvine, CA, USA) and was translated in steps of 0.5 mm for each scan across a distance of 50 mm.

**Figure 2:**
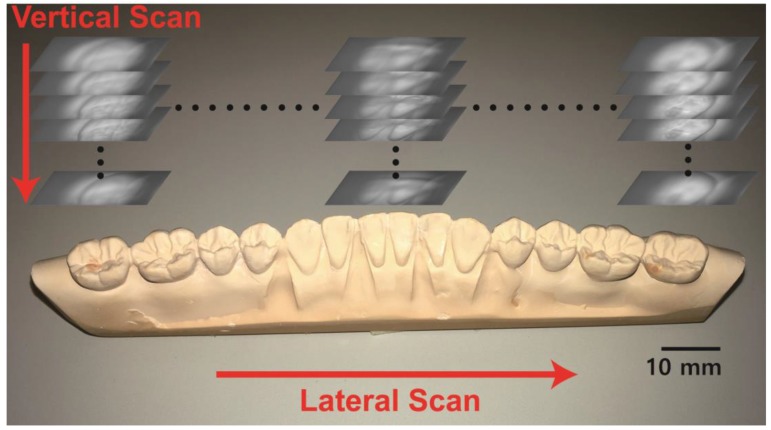
Picture of the dental cast (gypsum model) and the images captured by the scanning apparatus from the top view. Scanning process: Images of the dental cast were recorded with varying objective lens focal length (vertical scan). The dental cast moved laterally after each vertical scan was finished (lateral scan). Scale bar: 10 mm.

Figure 2 shows a picture of the gypsum dental cast and the scanning process for the reconstruction of a 3D model of the entire gypsum dental cast. We took top-view images of the dental cast with varying the focal length of the objective lens from the top to the bottom of the cast (vertical scan). After the vertical scan was complete, the dental cast was translated in the lateral direction with a 0.5 mm step (lateral scan), and the next vertical scan was performed. Each single scan along a straight line across the entire dental cast took approximately 190 s.

## 3. Results

**Figure 3:**
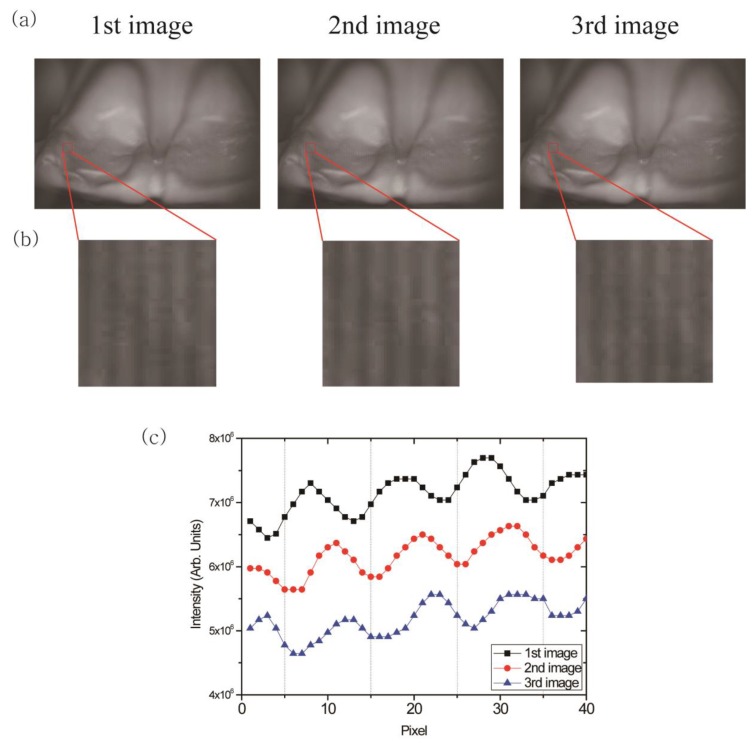
(**a**) Images (top row) of the dental gypsum cast and (**b**) magnified images (bottom row) of the in-focus area. Sinusoidal fringe patterns were visualized only within the in-focus areas. (**c**) The intensity profiles of the magnified images (Figure 3b) along the horizontal axis of the images. The intensity profiles were vertically translated for better discrimination. The sinusoidal fringe patterns were shifted by T/3 (T: periodicity of the patterns) between each image.

We performed optical sectioning using the recorded raw images of the dental cast. Figure 3a,b show the images and the magnified fragments of the dental cast from the same focal plane. Sinusoidal fringe patterns generated by the Ronchi ruling were overlaid onto the raw images of the dental cast. The sinusoidal fringe patterns were only visible within the in-focus area because the Ronchi ruling and the CMOS camera were located at the same distance from the imaging lens. The patterns were shifted by T/3 between each image, where T is the periodicity of the patterns (Figure 3c).

**Figure 4:**
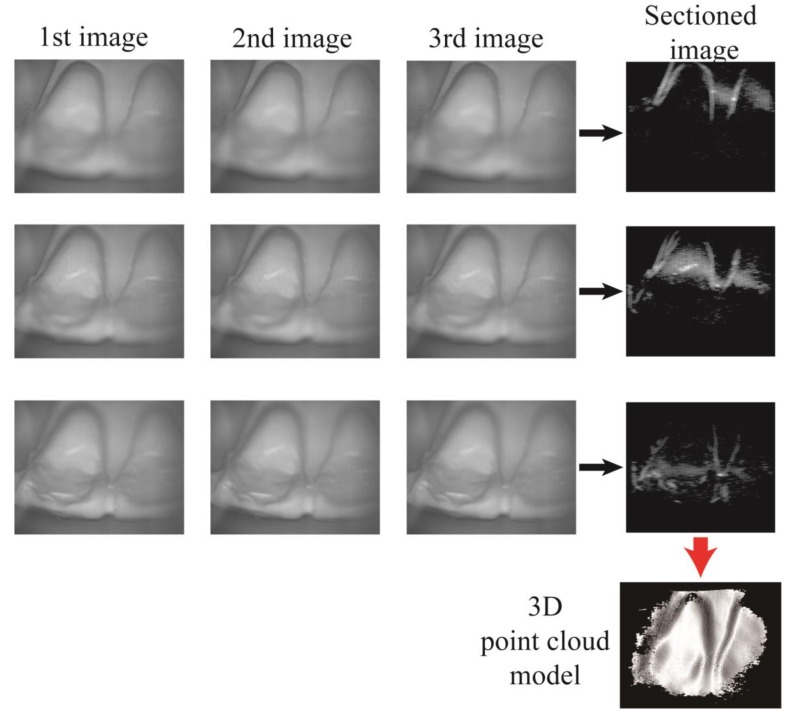
Optical sectioning from the raw images of the dental cast. The intensity of each pixel of the sectioned image is defined in Equation (1). The sectioned images from different focal planes were stacked to reconstruct a 3D point cloud model of the dental cast.

For the optical sectioning, we only used the intensity of each pixel of the raw images rather than the phase unwrapping method [35,36,37]. Sectioned images were obtained from the root-mean-square (RMS) of the sum of the squared differences between each raw image from the same focal plane. The modulation contrast of each pixel of the sectioned image, $I_{mn,sectioned}$, was defined as:

(1) $$I_{mn,sectioned}=\frac{1}{3}{\left\{{\left(I_{mn,1}-I_{mn,2}\right)}^{2}+{\left(I_{mn,2}-I_{mn,3}\right)}^{2}+{\left(I_{mn,3}-I_{mn,1}\right)}^{2}\right\}}^{\frac{1}{2}}$$

where $I_{mn,N}$ (N = 1,2,3) is the intensity of the pixel located at coordinate (*m,n*) of the Nth raw image. The raw images of the dental cast and its corresponding sectioned images are depicted in Figure 4. In the sectioned images, the white color represents the maximum-intensity of the optical sectioning and indicates a perfect in-focus state. The out-of-focus background in the sectioned images was removed by setting the upper threshold for the intensity modulation and by decoding the in-focus information. The lateral resolution of the sectioned images, which was determined by the periodicity of the sinusoidal fringe pattern, is 50 μm. We calculated the axial distance between each sectioned plane and the exit pupil of the objective lens, assuming that the tunable lens and the objective lens were a single optical element. We reconstructed 3D point clouds of the teeth based on the 3D coordinates and intensity data from the optical sectioning.

**Figure 5:**
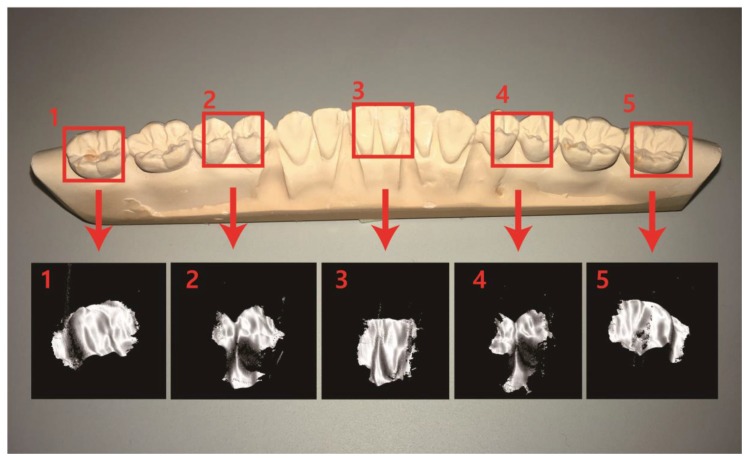
The dental cast and the 3D point cloud models of the dental cast from different scanning points. The sectioned images were combined in a single volume to show different areas of the dental cast.

Figure 5 shows a picture of the dental cast and the 3D point cloud models (polygonised surfaces) from different scanning positions. We combined sectioned images in a single volume to show different areas of the dental cast (see Movies in the online supplementary data to show rotating view of reconstructed 3D models of the dental cast). The lateral field of view (FOV) of the 3D scanning apparatus was 11.27 mm × 6 mm. During the background removal process, some in-focus information was sacrificed, which reduced the lateral area of the reconstructed 3D models. The optical sectioning depth was 10 mm, and we sliced 100 layers at an axial stepping interval of 100 μm. Since the axial stepping interval was much larger than the focal depth of the objective lens, the axial resolution of the 3D point cloud model was the same as the axial stepping interval.

**Figure 6:**
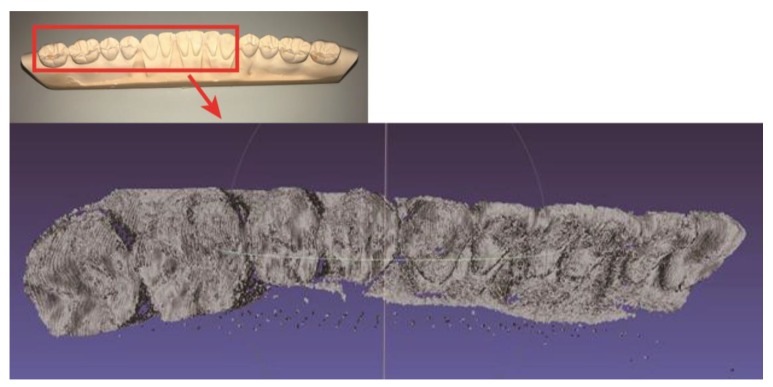
The 3D point cloud of the dental cast (half arch) reconstructed from 3D registration.

We performed 3D registration (3D model stitching) to reconstruct a 3D model of the dental cast, whose dimensions were beyond the FOV of the scanning apparatus. Multiple 3D point clouds from adjacent scanning points were stitched together based on distinctive features of the 3D point clouds. To reconstruct a 3D model of the dental cast from a sequence of 3D point clouds, the iterative closet point (ICP) algorithm [38] was used. The ICP algorithm performs pairwise registration in which it aligns the adjacent 3D point clouds. The implementation of ICP worked by iteratively finding corresponding points between two 3D point clouds and estimating the rigid transformation, which minimized the distance between these two corresponding point pairs in order to align the latter 3D point cloud with the former 3D point cloud. To compose the dental cast, the ICP algorithm was repeatedly used to process successively scanned 3D point clouds. The first point cloud was used to establish the reference coordinate system. Then, each 3D point cloud was transformed to the reference coordinate system, where the transformation was a multiple of the pairwise transformation. Figure 6 shows the 3D point cloud model of the dental cast reconstructed from the 3D registration based on the Point Cloud Library [39].

## 4. Discussion

We demonstrated a 3D dental scanning apparatus based on structured illumination, and suggested a simple algorithm for 3D reconstruction. In addition, we performed 3D registration of the 3D point cloud models of each scanning point to build a 3D model of the dental gypsum cast. We constructed a fast and compact 3D scanning apparatus with a liquid tunable lens and piezomotor stage. The elapsed time for a full scan was approximately 190 s, and the physical dimensions of the apparatus were 275 mm × 176 mm × 72 mm. With this 3D scanning apparatus, we reconstructed 3D point cloud models of a dental gypsum cast. The axial resolution of the 3D point cloud models was 100 μm, which coincides to the axial step of the tuning of the working distance of the 3D scanning apparatus. The accuracy of a commercial 3D intraoral scanner, which is based on the stereovision technique (Cerec Omnicam, Dentsply Sirona, York, PA, USA), was reported to be 149 μm [40]. The accuracy of a commercial intraoral scanner which is based on structured illumination (Trios 3, 3shape) was not reported officially yet. At present, the scanning speed for our 3D scanning apparatus is restricted by the sensitivity and speed of the CMOS camera. It is possible to make the scanning faster with more sensitive and faster custom-built CMOS sensors. For the scanning speed of the commercial 3D intraoral scanners, it took 4:18 minutes to scan a full arch for Cerec Omnicam and 30 s for Trios 3. However, Trios 3 used a CMOS sensor of 3000 frames per second and is currently the most expensive intraoral scanner. We expect our results will contribute to the development of a faster and more precise 3D intraoral scanning apparatus. Moreover, our research will pave the way for further investigation of non-contact 3D shape measurements

## Supplementary Materials

The following are available online at http://www.mdpi.com/1424-8220/17/7/1634/s1, Video S1–S3: rotating view of reconstructed 3D models of the dental cast.
